# 

**Published:** 1905-07

**Authors:** 


Harpooning the Devil Fish. }

VOL. XXI. JULY, 1905. No. 1.

INDEXED
S. G. O.

### TEXAS Medical Journal.

TWENTY-FIRST YEAR.

SUBSCRIPTION, $1.00 A YEAR, IN ADVANCE.

SINGLE COPIES, 10 CENTS.

SURGEON GENERAL'S OFFICE
JUL. 31-1905

PUBLISHED AT AUSTIN, TEXAS, BY

F. E. DANIEL, M. D.,

PROPRIETOR.

PRINTED BY THE VON BOECKMANN-JONES CO.

Entered at the Postoffice at Austin, Texas, as Second-class Mail Matter.

